# New Views of the Nature and Treatment of Syphilis

**Published:** 1846-07

**Authors:** 


Art. II.—*Lettre sur la Syphilis ou Vues Nouvelles sur la Nature, et le Traitement de la Maladie Vénérienne.* Par F. S. Ratier.—*Paris,* 1846.

*New Views of the Nature and Treatment of Syphilis.* By F. S. Ratier. —*Paris,* 1846. 8vo, pp. 100.

We cannot discover in this brochure of M. Ratier that the contents in any way correspond with the title. Our author admits one species of primary sore, and one variety of constitutional disease only (the papular eruption) as characteristic of syphilis properly so called. These are considered specific, and the treatment recommended is that which English surgeons are commonly in the habit of employing. Other symptoms the result of sexual intercourse, as the various forms of gonorrhœa, balanitis, vegetations, bubos, &c. &c., are due to a variety of causes, but are not purely specific, nor is the treatment suited to the first forms at all applicable to the second. In all this there is nothing to detain us. We are sorry we cannot extract from M. Ratier's letter anything either in reference to the pathology or treatment of venereal diseases new or useful to the English reader.

